# Neuroglobin, a Novel Target for Endogenous Neuroprotection against Stroke and Neurodegenerative Disorders

**DOI:** 10.3390/ijms13066995

**Published:** 2012-06-07

**Authors:** Zhanyang Yu, Ning Liu, Jianxiang Liu, Kevin Yang, Xiaoying Wang

**Affiliations:** 1 Neuroprotection Research Laboratory, Department of Neurology and Radiology, Massachusetts General Hospital, Neuroscience Program, Harvard Medical School, Room 2401/2411A, 149 13th Street, Charlestown Boston, MA 02129, USA; E-Mails: liuning0731@yahoo.cn (N.L.); kevin.yang95@gmail.com (K.Y.); 2 National Institute for Radiological Protection, China Center for Disease Control and Prevention, Beijing 100088, China; E-Mail: jxlamy@gmail.com

**Keywords:** neuroglobin, neuroprotection, stroke, neurodegenerative diseases, hypoxia/ischemia, high-throughput screening

## Abstract

Brain neurons and tissues respond to sublethal injury by activating endogenous protective pathways. Recently, following the failure of a large number of clinical trials for protective strategies against stroke that aim to inhibit a specific ischemia response pathway, endogenous neuroprotection has emerged as a more promising and hopeful strategy for development of therapeutics against stroke and neurodegenerative disorders. Neuroglobin (Ngb) is an oxygen-binding globin protein that is highly and specifically expressed in brain neurons. Accumulating evidence have clearly demonstrated that Ngb is an endogenous neuroprotective molecule against hypoxic/ischemic and oxidative stress-related insults in cultured neurons and animals, as well as neurodegenerative disorders such as Alzheimer’s disease, thus any pharmacological strategy that can up-regulate endogenous Ngb expression may lead to novel therapeutics against these brain disorders. In this review, we summarize recent studies about the biological function, regulation of gene expression, and neuroprotective mechanisms of Ngb. Furthermore, strategies for identification of chemical compounds that can up-regulate endogenous Ngb expression for neuroprotection against stroke and neurodegenerative disorders are discussed.

## 1. Introduction

In the past 20 years, a large number of clinical trials for neuroprotectants against stroke and neurodegenerative disorders have yielded mostly disappointing outcomes. Nevertheless, enormous knowledge has been learnt from these trials and related basic investigations. In particular, preconditioning studies have demonstrated that activation of endogenous protective mechanisms can prevent or limit brain damage. Activation of these endogenous protective mechanisms could be a more promising strategy for the development of new therapies against stroke and neurodegenerative disorders. Neuroglobin (Ngb) is an oxygen-binding globin protein that is highly and specifically expressed in brain neurons[1]. Accumulating evidence has proved that Ngb is an endogenous neuroprotective molecule against hypoxic/ischemic insults in cultured neurons and animals. Enhanced Ngb gene expression inversely correlates with the severity of histological and functional deficits after ischemic stroke [2–5], while Ngb knock-down deteriorates the outcome of hypoxic/ischemic brain injury [6]. Furthermore, Ngb overexpression is also protective against beta-amyloid-induced neurotoxicity and transgenic Alzheimer phenotype *in vivo* [7]. These findings strongly suggest that pharmacological strategies that can up-regulate endogenous Ngb expression may be developed into a novel therapeutic approach for stroke intervention. Here we review the recent experimental findings from our laboratory and others about Ngb’s biological function, regulation of gene expression, and neuroprotection mechanisms. Additionally, we discuss strategies to identify chemical compounds that can up-regulate endogenous Ngb expression for neuroprotection against stroke and neurodegenerative disorders.

## 2. Ngb Can Serve as a Target for Development of Therapeutics against Stroke and Neurodegenerative Disorders

Endogenous protection pathways can be activated in the brain in response to a wide variety of stimuli, in which a large number of proteins are involved and show protective effects [8]. Endogenous neuroprotective molecules, including transcription factors, have been considered new therapeutic targets against stroke or related disorders [9]. Since elevated levels of these molecules are related to improved physiological outcomes, strategies that can up-regulate the expression of these molecules are expected to be neuroprotective.

One of the endogenous neuroprotective molecules is Ngb, whose broad neuroprotective effects against stroke and neurodegenerative disorders have been demonstrated by a series of experimental studies [10–14]. Since Ngb is an intracellular protein that normally does not cross cell membranes, except in zebrafish [15], delivery of exogenous Ngb protein is generally considered unfeasible as a therapy, especially for CNS disorders such as stroke, thus seeking small molecules capable of up-regulating endogenous Ngb may lead to the development of new approaches for the treatment of stroke and related disorders. Indeed, a couple of groups have recently reported that Ngb can be up-regulated by a few chemical compounds, including valproic acid, cinnamic acid and 17β-estradiol [16,17], which is a good start to develop Ngb-targeted therapeutics against stroke and neurodegenerative disorders. In addition, the discovery of these preliminary Ngb stimulators may serve as positive controls in the future establishment of a larger scale screening system for Ngb-stimulating compounds.

## 3. Neuroprotective Roles of Ngb against Hypoxic/Ischemic and Oxidative Stress-Related Brain/Neuron Injury

Globins, including hemoglobin, myoglobin and cytoglobin, are oxygen-binding proteins that widely exist and play important roles in many taxa, including bacteria, plant, fungi and animals. In 2000, Ngb was for the first time identified as a new globin family member that is highly expressed in brain neurons [1]. Ngb is evolutionally highly conserved, with mouse and human Ngb sharing 94% identity in protein sequence. Besides brain neurons, Ngb is also highly expressed in the peripheral nervous system, endocrine tissues and retina [18]. Since the discovery of Ngb, a large array of experimental studies have approved its neuroprotective functions and looked into possible underlying mechanisms, which have been extensively summarized in several review articles [13,19–24].

The oxygen-binding property and neuron specific expression of Ngb are strong indications of Ngb’s neuroprotection role against hypoxic/ischemic neuron injury. Gene expression alteration approaches have been applied to address this issue. The first report in this category by Sun *et al.* showed that antisense-mediated knock-down of Ngb rendered cortical neurons more vulnerable to hypoxia, whereas Ngb overexpression conferred protection of cultured neurons against hypoxia [2]. A similar effect was observed in the neuroblastoma cell line SH-SY5Y, that Ngb over-expression enhanced cell survival under conditions of anoxia or oxygen and glucose deprivation (OGD) [25]. In animal stroke models, Ngb-overexpression by adeno-associated virus administration significantly reduced infarct size in rats following middle cerebral artery occlusion (MCAO), and the outcome was reversed when Ngb was knocked down using anti-sense oligonucleotide [6]. In the Ngb-overexpressing transgenic (Ngb-Tg) mice, Dr. Greenberg’s group found that the cerebral infarct size after MCAO was reduced by approximately 30% compared to wild type [26]. Our lab also tested the neuroprotective effects of Ngb in transient focal cerebral ischemia using our own Ngb-overexpressing transgenic mouse line [5]. Our results were broadly consistent with the study by Dr. Greenberg’s group [26], and further documented that the reduction of brain infarction in Ngb-overexpressing transgenic mice can be sustained up to 14 days after ischemia compared to wild type controls, suggesting that Ngb overexpression is neuroprotective against transient focal cerebral ischemia, but the involved mechanisms need to be further characterized. It should be noted that the above experiments with Ngb transgenic approaches are “outcome” studies. These findings are very informative about the effects of artificially increased Ngb level on stroke, but have limitations to fundamentally define or interpret the role of endogenous Ngb, thus in the future, a neuron-specific and inducible Ngb knockdown approach would be very useful in further investigations of Ngb function in the normal, *versus* ischemic, brain.

Previous studies on the neuroprotective effects of Ngb are mostly based on models using transgenic overexpression approaches. However, for stroke therapy, it would be more practical and easily applicable if increased Ngb protein level in brain tissue can be achieved after stroke. A recent report by Cai *et al.* [27] presented important progress toward this direction. They delivered Ngb protein into mouse brain tissue using the 11-amino-acid human immunodeficiency virus trans-activator of the transcription (TAT) protein transduction domain. The results showed that the brain tissue was protected from ischemic stroke by both pre-treatment of TAT-Ngb or post-treatment right after reperfusion onset in a mild MCAO mouse model, but no beneficial outcome was observed if the TAT-Ngb was administered 2 hr after ischemia onset. This study suggests that Ngb-overexpression might be beneficial for early stroke treatment, and for stroke prevention for individuals with higher stroke risk as well.

## 4. Neuroprotective Effects of Ngb against Neurodegenerative and Other Neurological Disorders

As a brain specific oxygen-binding protein, it is not surprising that Ngb is also protective against other models of neurological disorders. One example is that Ngb overexpression was protective against beta-amyloid and NMDA toxicity in both cultured neurons and in the Alzheimer’s disease (AD) model of mice [7,28]. Additionally, Ngb expression can be up-regulated in the cerebellum of rat pups exposed to maternal epileptic seizures, implying that Ngb may also be protective against seizures [29]. Our recent study showed that Ngb overexpression protects retinal ganglion cells (RGC) against ocular hypertension and glaucomatous damage [30]. The broader implication of Ngb in neurodegenerative disorders indicates the importance of Ngb as an endogenous neuroprotective molecule, therefore any strategy that can up-regulate endogenous Ngb expression is more likely to be clinically beneficial.

## 5. Molecular Mechanisms of Ngb Neuroprotection

It has been widely accepted that Ngb is protective against hypoxic/ischemic brain injury [5,26], however, the underlying mechanisms remain poorly defined. Initial evidence suggests that the neuroprotective effect of Ngb may be largely linked to its structural features in O_2_ and NO binding. Furthermore, putative signal transduction and mitochondrial function preservation may also be involved in the protective mechanisms.

### 5.1. Oxygen Sensing and ROS Scavenging by Ngb

Ngb protein in human or mouse exists as a monomer, which is distinct from the heterotetrameric hemoglobins. The 3D structure of human [31] or mouse Ngb [32] has been solved, showing that the heme is inserted into the protein in two different orientations. The lack of orientation selectivity is possibly related to the presence of a large cavity lining the heme and to the increased mobility of heme contacts [32]. Human Ngb displays a typical globin fold, and the heme-iron is hexacoordinate [33], with proximal HisF8 and distal HisE7 that provide the two axial coordination bonds. An elongated protein matrix cavity in the 3D structure would facilitate O_2_ diffusion to the heme [34]. Ngb was originally thought to function in O_2_ storage and transportation, however, since Ngb has a very high O_2_ binding rate and low O_2_ dissociation rate, and Ngb protein concentration in the brain is relatively low (~1 μM) [20], thus O_2_ storage and transportation might not be Ngb’s main function, instead, Ngb may function in O_2_ sensing [35,36].

A number of studies have indicated that Ngb’s neuroprotection role is related to its ability in scavenging reactive species, because Ngb can directly bind to nitric oxide (NO) with high intrinsic affinity and a low dissociation rate [37]. In support of this function, a high degree of co-localization of neuronal nitric oxide synthase (nNOS) and Ngb has been detected within anterior basomedial amygdala (BMA), lateral hypothalamus and laterodorsal tegmental nucleus (LDTg) [38], implying that in these neurons, NO could be the endogenous ligand for Ngb. Furthermore, Brunori *et al*. [39] found that the oxygenated derivative of Ngb, Ngb-O_2_, reacts with NO rapidly to produce NO^3−^ and met-Ngb. This pathway would dispose of NO and may in turn protect cellular respiration jeopardized by the inhibitory effect of NO on cytochrome *c* oxidase activity [40,41]. Additionally, Ngb overexpression rendered HN33 neuroblastoma cells more resistant to NO-induced cell death compared to wild type cells, suggesting the ability of Ngb in neutralizing the neurotoxic effects of reactive nitrogen species [42]. Ngb was also shown to be protective against other oxidative challenges in cultured neurons. For example, Ngb-overexpression conferred protection in SH-SY5Y cells directly injured by H_2_O_2_ [43]. Furthermore, beta-amyloid-induced cytotoxicity to PC12 cells, marked by reactive oxygen species production and lipids peroxidation, was ameliorated by Ngb overexpression [44]. All of these findings suggest that Ngb may have the function of reactive oxygen species scavenging.

### 5.2. Regulation of Signal Transduction

In addition to the possible O_2_ sensing and ROS scavenging functions described above, Ngb has also been hypothesized to act as a signal transducer. Dr. Morishima’s lab found that ferric human Ngb (met-Ngb) binds to the GDP-bound state of G protein α subunit (Gα), and exerts guanine-nucleotide dissociation inhibitor (GDI) activity [45]. Ferric Ngb inhibits the exchange of GDP for GTP, thus prevents the Gα subunit from binding to the Gβγ complex and activates the downstream signal transduction pathway, which is protective against oxidative stress [46]. This hypothesis was additionally supported by the observation that the guanine-nucleotide dissociation inhibitor activity of human Ngb is required for its neuroprotection for PC12 cells under oxidative stress [47].

Khan *et al.* have shown that Ngb binds two members of the Rho GTPase family, Rac1 and Rho A, as well as the Pak1 kinase, a key regulator of actin assembly and Rho-GDI-GTPase signaling complex activity under hypoxia [48]. They hypothesized that Ngb may play a neuroprotective role by inhibiting the dissociation of the GTPase Rac-1 from its endogenous GDI, which can reduce hypoxia-induced actin polymerization and microdomain aggregation. Moreover, Ngb was also found to interact with other targets, such as flotillin-1 (a lipid raft microdomain-associated protein) [49] and cystatin C (a cysteine protease inhibitor) [50], the latter proposing a possibility that Ngb modulates the intracellular transport of cystatin C to protect against neuronal death caused by oxidative stress.

Based on the above findings and the kinetics study of Ngb reaction with O_2_ and NO, Brunori *et al*. proposed that Ngb might function as a sensor of the relative O_2_ and NO concentrations in the tissue [39]. Supportive evidence for this hypothesis is that Ngb oxygenation is quickly reversible, and the oxygenated derivative, Ngb-O_2_, reacts rapidly with NO to produce NO^3−^ and met-Ngb. This process competes effectively with the direct formation of Ngb-NO, which excludes the production of met-Ngb, and its protective signaling function as a guanine-nucleotide dissociation inhibitor [45]. On the other hand, Tsio *et al*. [51] reported that human Ngb can function as a redox-regulated nitrite reductase as deoxygenated human Ngb can convert nitrate to NO. These studies suggest that Ngb may function as a physiological oxidative stress sensor and may regulate intracellular hypoxic NO-signaling pathways.

The proper function of Ngb requires a met-Ngb reductase to maintain the balance between redox and oxidized Ngb. Complying with this scenario, Dr. Brunori’s lab found that NADH:flavorubredoxin oxidoreductase (FlRd-red) from *E. coli* is able to slowly reduce Ngb at catalytic concentrations [52]. More interestingly, *E. coli* FlRd-red was found to share significant similarity with human apoptosis-inducing factor (AIF), the principal mediator of the so-called caspase-independent programmed cell death [53]. In healthy cells, AIF is located within the mitochondrion, but upon permeabilization of mitochondrial outer membrane, it translocates first to the cytosol and then to the nucleus, where it triggers chromatin condensation followed by massive DNA fragmentation [53]. It is therefore possible that AIF may reduce cytoplasmic met-Ngb on its way from the mitochondrion to nucleus, and depending on O_2_ tension, the reduced Ngb can either interfere with the classical apoptotic pathway by reducing ferric Cytochrome c (Cyt c) [54] or become involved in NO scavenging [39].

Previous studies have suggested that Ngb is very likely to function in multiple pathways leading to its neuroprotection role, as described above. Based on this, our laboratory has hypothesized that Ngb may also play a role in regulating gene expression in response to hypoxia/OGD; we therefore performed a microarray screening to examine the effect of Ngb overexpression on the expression of hypoxic-response genes in mouse cortical neurons. We found that 20 genes were downregulated at early phase OGD/Reoxygenaton in wild type neurons, while 12 of them were no longer significantly changed in Ngb-overexpressing neurons [55]. These genes are broadly involved in neuronal function and survival, indicating that Ngb may play roles in multiple cell survival signaling pathways.

Another example of Ngb involvement in signal transduction was found in the animal model of Alzheimer’s Disease (AD). Ngb has been shown to attenuate beta-amyloid-induced neurotoxicity [7]. As a potential molecular mechanism involved in this effect, it was found that Ngb overexpression attenuates tau hyperphosphorylation, a characterized pathological hallmark of AD brains, probably through activating the Akt signaling pathway [56]. One should note that almost all of these tests are “correlation” studies; the role of Ngb in these signaling pathways might be direct or indirect. Direct and causative evidence is still largely lacking. Nonetheless, Ngb has been demonstrated to be protective in stroke, AD, and could serve as a therapeutic target.

### 5.3. Maintenance of Mitochondrial Function

Ngb expression has been shown to be confined to metabolically active, oxygen-consuming cell types [21]. At the subcellular level, Ngb is associated with mitochondria and thus linked to the oxidative metabolism [57]. Mitochondria play key roles in energy production, ROS homeostasis, and cell death signaling. Mitochondria respond to various insults to cells, and its dysfunction is associated with a large variety of clinical phenotypes. It has been demonstrated that mitochondria comprises a central locus for energetic perturbations and oxidative stress in hypoxia/ischemia [58,59]. Experimental studies have shown that overexpression of Ngb promotes cell survival of PC12 cells against beta-amyloid toxicity and attenuates beta-amyloid-induced mitochondrial dysfunction [60]; and eliminates hypoxia-induced mitochondrial aggregation and neuron death [48]. Our lab also demonstrated that Ngb overexpression improves mitochondrial function and reduces oxidative stress after hypoxic insult in cultured mouse cortical neurons [61]. We found that at earlier time points after hypoxia/reoxygenation, no difference in neurotoxicity was observed between Ngb-overexpressing and wild type neurons, whereas the rates of decline of several mitochondria function biomarkers, including ATP level, 3-(4,5-dimethyl-2-thiazolyl)-2,5-diphenyl-2*H*-tetrazolium bromide (MTT) reduction, and mitochondrial membrane potential, were significantly ameliorated in Ngb-overexpressing neurons compared to wild type. At a later time point there was a significant reduction of neurotoxicity in Ngb-overexpressing neurons. Furthermore, Ngb overexpression reduced superoxide anion generation after hypoxia/reoxygenation, but glutathione levels were significantly improved, compared to wild type controls. Our data suggested that Ngb might affect both mitochondrial function and free radical generation as its potential neuroprotective mechanisms. However, there are multiple and probably inextricable feedback loops between preservation of mitochondrial energetics *versus* direct free radical scavenging [59,62–64]. We acknowledge that it will likely be impossible to unequivocally separate mitochondrial effects *versus* reactive oxygen species effects of Ngb.

Hypoxia and OGD result in mitochondrial depolarization [65]. The mitochondrial permeability transition pore (mPTP) is a protein pore formed across the inner and outer membrane of the mitochondria under pathological conditions such as stroke. In response to hypoxia/ischemia, mPTP opening caused release of Cyt c from mitochondria to cytosol [66], followed by activation of caspase-dependent or -independent apoptosis pathways [67–69]. Studies in our lab have shown that Ngb overexpression is correlated with reduced mPTP opening, and decreased Cyt c release as well (unpublished data). This suggests an inhibitory role of Ngb in OGD-induced mPTP opening, which has been thought to be one of the major causes of cell death in a variety of tissue ischemic damage scenarios, as occurs in heart attack and stroke. Thus Ngb inhibitory effect in mPTP opening may be an important mechanism of Ngb neuroprotection.

**Figure 1:**
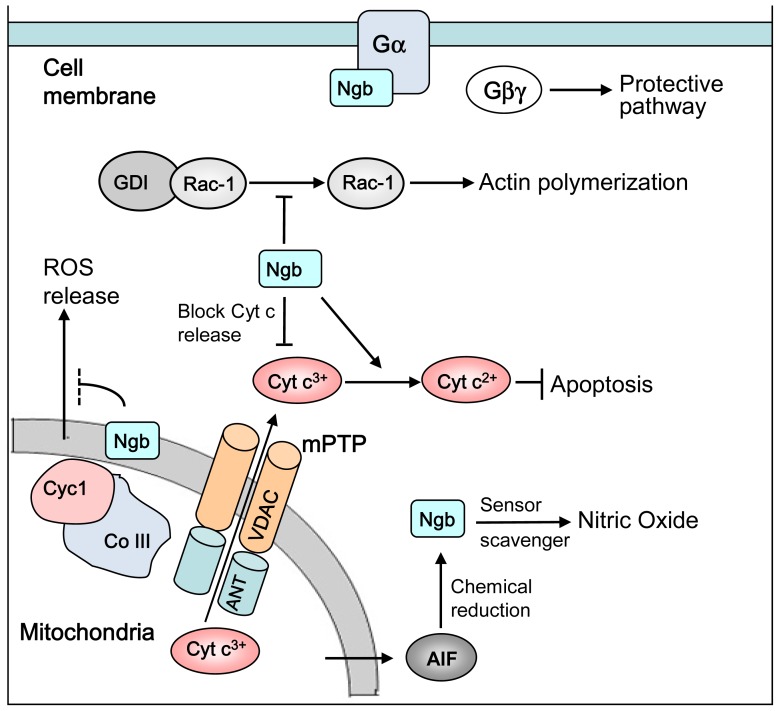
Possible molecular mechanisms of Ngb neuroprotection by modulating cell death/survival signaling pathways. Ngb may function as an O_2_ and NO sensor. Ngb has guanine-nucleotide dissociation inhibitor (GDI) activity and can prevent Gα from binding to the Gβγ complex and activates the downstream signaling pathway. Ngb may inhibit the dissociation of Rac-1 from its endogenous GDI, thus preventing hypoxia-induced actin polymerization and microdomain aggregation. Ngb may interact with VDAC and inhibit hypoxia/OGD-induced mPTP opening and Cyt c release from mitochondria. Ngb could convert Cyt c^3+^ to Cyt c^2+^, and subsequently interfere with apoptotic signaling cascades or scavenge nitric oxide. Ngb may interact with Cyc1 and inhibit hypoxia/OGD-induced ROS generation by mitochondria complex III.

To further dissect the molecular mechanisms of Ngb neuroprotection, our laboratory recently did a screening for the protein interaction partners of mouse Ngb, using yeast two-hybrid assay. We identified several Ngb-binding proteins, including Na/K ATPase beta 1, cytochrome c1, ubiquitin C, voltage-dependant anion channel (VDAC) and a few more [70]. Among these Ngb-binding protein candidates, some are biologically important for neuronal function and survival. For example, cytochrome c1 is a subunit of the cytochrome bc1 complex (mitochondria complex III), which plays an important role in mitochondria function for energy transduction and generation of the superoxide anion [71], and it also plays pathological roles in response to oxidative stress [72,73] and regulates hypoxia-inducible-factor-1 activation induced by hypoxia [74–76]. Cytochrome c1 is localized in the intermembrane space between the outer and inner membrane of mitochondria [77]. The mitochondrial outer membrane contains the protein “porin”, which forms an aqueous channel through which proteins up to 10 kd can pass and go into the intermembrane space. It has been known that hypoxia-induced superoxide, as well as apoptotic signaling molecules, such as Bax, may cause permeabilization of the mitochondrial outer membrane [78,79]. Thus, as a 16 kd monomer, Ngb might be able to pass the outer membrane to bind and affect the function of cytochrome c1. Additionally, results from our laboratory showed that Ngb overexpression is able to decrease OGD-induced mitochondria permeability transition pore (mPTP) opening and Cyt c release from mitochondria. The interaction between Ngb and VDAC, an mPTP component, is supportive of Ngb’s effect in mPTP. However, the phenomenon of Ngb binding to other proteins and binding status-correlated alteration of cell signaling and mitochondrial function requires further investigation. The major hypotheses about Ngb-involved signaling pathways are summarized in Figure 1.

## 6. Tissue Specific Expression of Ngb Gene and Its Regulation

### 6.1. Neuron-Specific Expression of Ngb Gene

From the beginning of its discovery, Ngb gene expression has been found to be highly tissue-specific. *In situ* hybridization showed that Ngb mRNA was widely distributed throughout the adult rat brain, including cerebral cortex, hippocampus, and subcortical structures such as thalamus, hypothalamus, olfactory bulb, and cerebellum [33,80,81]. The distribution of Ngb protein is consistent with its mRNA localization, and the subcellular immunoreactivity is restricted to the cytoplasm.

Among all Ngb-expressing cells, the highest expression is seen in the retina, with the estimated concentration about 100-fold higher than in the brain [82]. Ngb mRNA was detected in the perikarya of the nuclear and ganglion layers of the neuronal retina, whereas the protein was present mainly in the plexiform layers and in the ellipsoid region of the photoreceptor inner segment [83]. The distribution of Ngb correlates with the subcellular localization of mitochondria and with the relative oxygen demands. These findings suggest that Ngb supplies oxygen to the retina, similar to myoglobin in the myocardium and skeletal muscle. Although Ngb concentration in the brain is relatively lower than in the retina, considering that the neuron is the major cell type in the brain specifically expressing Ngb, thus Ngb may be a unique molecule that plays certain roles in maintaining normal neuron function and protectively responding to pathological insults.

### 6.2. Ngb Gene Expression under Pathological Conditions

The expression of Ngb gene is up-regulated under hypoxic/ischemic conditions both in cultured cells [84,85] and in stroke animal brains [2,6,86,87]. Ngb is also up-regulated in the cerebellum of mouse pups in response to hypoxic-ischemic insults caused by maternal seizures during intrauterine life [29]. These data imply that Ngb up-regulation could be an endogenous compensatory or protective mechanism in response to the sublethal hypoxic/ischemic insults to brain neurons. More importantly, a recent report showed that Ngb expression is increased in the cortical peri-infarct region in stroke patients, suggesting its clinical relevance for endogenous neuroprotection [88]. In contrast to acute hypoxic conditions, a chronic hypoxia (10% oxygen for 14 days) did not increase Ngb gene expression in mRNA or protein level in the mouse [89]. Moreover, two-hour exposure of mice to 7.6% oxygen did not up-regulate brain Ngb either [83]. However, others reported opposite results that housing rats in 10% oxygen for up to 14 days up-regulated Ngb mRNA in the rat brain [90]. Thus, there might also be species- and condition-dependent differences for Ngb responses to hypoxic conditions. In addition to hypoxia/ischemia, aging might also be an influential factor for Ngb expression. Sun *et al*. have demonstrated that Ngb expression level decreased to about a half in aged rats (24 months) compared to young ones (3, 12 months) in various regions of brain, implying the pathophysiological importance of Ngb in age-related neurodegenerative diseases [44].

### 6.3. Transcriptional Regulation of Ngb Gene

A few recent studies focused on the transcriptional regulation mechanisms of Ngb gene expression. Zhang *et al.* reported that transcription factors Sp1 and Sp3 can bind to the human Ngb promoter region and are responsible for transactivation of Ngb promoter [91]. Our lab has analyzed the core promoter region of the mouse Ngb gene, and further characterized the transcription factors required for Ngb gene expression under both resting and hypoxic conditions [92]. We located the core promoter of mouse Ngb gene to the 554 bp segment before the transcription start codon. Complementary approaches have demonstrated that transcription factors, NFκB family members (p65, p50, cRel), Egr1, and Sp1 bind the Ngb promoter, and are responsible for basal Ngb expression. Moreover, NFκB (p65) and Sp1, as well as Hif1α, were also responsible for hypoxia-induced up-regulation of Ngb expression. These findings will be helpful in establishing strategies to screen for compounds that can up-regulate endogenous Ngb gene expression.

### 6.4. Ngb Gene Expression in Non-Neuronal Cells

Besides brain neurons and the retina, Ngb expression could also be detected in other tissues such as pancreas, adrenal gland and testes, but the expression level was lower [93]. It was reported that Ngb mRNA is detectable in cultured astrocytes from newborn mouse brain, and that Ngb protein co-localizes with glial fibrillary acid protein (GFAP) in cultured astrocytes [94]. Moreover, a number of studies have demonstrated that Ngb was expressed in various tumor tissues, including glioblastoma [95,96] and astrocytoma [97]. These findings imply a broader involvement of Ngb under pathological conditions in different tissues, and suggest Ngb may be related to the adaptation of tumor cells to hypoxic microenvironments, which is consistent with its role in oxygen sensing and hypoxic signaling.

Overall, emerging data have clearly documented that up-regulation of Ngb gene expression is neuroprotective against hypoxic/ischemic brain injury [5,26], therefore targeting Ngb for endogenous neuroprotection would be translationally significant. We need to seek strategies to identify small molecules that elevate endogenous Ngb, which would help us in the development of new therapy approaches for stroke and other related neurological disorders.

## 7. Targeting Ngb for Endogenous Neuroprotection

### 7.1. Previously Identified Chemical Compounds that Up-Regulate Endogenous Ngb Expression

As described earlier (Section 2), a few chemical compounds have been identified that are able to up-regulate endogenous Ngb gene expression, including valproic acid (VPA), cinnamic acid and 17β-estradiol (E2) [16,17]. VPA and cinnamic acid were identified in a small-scale screening for Ngb up-regulators in cultured neuroblastoma cell line HN33 [16]. Interestingly, earlier studies have already shown that VPA was protective in rodent models of cerebral ischemia [20,22]. VPA is a commonly used drug to treat seizures and bipolar mood disorder, which at least in part warrants its clinical safety. However, previous studies suggested that the neuroprotective effect of VPA was through its function in histone deacetylase (HDAC) inhibition, and it is unclear whether Ngb induction plays a role in VPA neuroprotection. Cinnamic acid is a natural substance obtained from cinnamon oil. Cinnamic acid reduced glutamate toxicity in primary cultured rat cortical neurons [27], but did not protect GT1-7 (immortalized mouse hypothalamic) cells from oxygen and glucose deprivation (OGD) [28] *in vitro*. Another study also showed negative result of cinnamic acid in protection against beta-amyloid-induced toxicity in a rat-derived neuronal cell line [98]. These studies cast doubt on the neuroprotective effect of cinnamic acid in animal stroke models, which nevertheless is worth investigating in the future.

17β-estradiol (E2) was found to upregulate Ngb expression in both the SK-N-BE human neuroblastoma cell line and mouse hippocampal neurons [17]. As a type of estrogen, E2 has been reported to be protective for neuronal cells against a variety of insults, including H_2_O_2_ [99,100] and oxygen-glucose deprivation [101]. E2 can also attenuate the toxicity of beta-amyloid and glutamate in a hippocampal cell line [102]. In addition, estrogen therapy in post-menopausal women is associated with decreased incidence and enhanced recovery from ischemic stroke [103]. However, a serious drawback of E2 is its potential in carcinogenesis. E2 exposure to postmenopausal women is associated with an increased risk of endometrial and breast cancer [104,105]. Animal studies further confirmed the tumorigenesis caused by E2 administration [106,107]. Thus the prospect of E2 as a neuroprotective reagent looks slim.

It should be noted that the Ngb-upregulation effect of the above three molecules were all discovered in neuron cultures, thus many issues remain to be investigated, e.g., their Ngb regulation effect in animal models, the function and specificity of Ngb regulation. These three molecules need to be further studied as to whether they can be developed into therapeutic molecules via up-regulating Ngb. We expect that more small molecules may be Ngb upregulators and additional screening is necessary. As the availability of positive controls in screening assay is critical, it is necessary to justify in different models whether VPA, cinnamic acid and E2 can serve as positive controls for future screening to identify more Ngb upregulators.

### 7.2. Establishment of a Cell-based High Throughput Screening System for Identification of Small Molecules Capable of Ngb Upregulation

Our laboratory has just started to establish a cell-based high throughput screening (HTS) system for identifying novel small molecules capable of up-regulating Ngb expression. This screening approach has been successfully used in searching compounds for up-regulating intracellular proteins [108]. For example, activation of NF-κB has been targeted in a cell-based high-throughput screening to identify neuroprotectants [9]. A number of identified small molecules are being tested in preclinical animal studies, some of them initially showed promising results [109–111].

Generally, HTS incorporates two steps: (1) Establishment of a biological detection system; (2) Screening and validation of effective compounds. A common method of biological detection for transcriptional activation is to employ a cell-based assay with a reporter gene driven by the key promoter elements of the target gene. A popularly applied reporter gene is the firefly luciferase gene, due to its high sensitivity and cost efficiency [108,112]. In practice, the very first task before establishing the detection system is to identify the promoter of the target gene and then develop a reporter construct with this promoter. For Ngb, one study has identified a 2.0 kb promoter region for human Ngb gene [113]. Our recently published study further located the core promoter region of mouse Ngb gene to the 554 bp fragment before the transcription start codon [92]. This promoter region will be used to make luciferase reporter constructs.

A standard drug development procedure goes from cell assay to animal model, and eventually to clinical trial. An ideal compound would be able to induce the expression of endogenous neuroprotective molecules in both animal and human neurons and brains, therefore it is necessary to establish both mouse and human cell-based reporter constructs, *i.e.*, Ngb promoter-driven luciferase construct, for Ngb up-regulation compound screening, and to subsequently generate stable cell lines containing this Ngb reporter construct [110].

Once the stable cell lines containing Ngb reporter construct has been validated, compound screening can be performed on a cell-based high throughput assay system [114]. The identified compounds that can up-regulate Ngb expression should then be validated as follows: (1) Only the compounds that can up-regulate both mouse and human Ngb promoter activities will undergo further investigations; (2) To determine whether changes of Ngb reporter activities truly reflect endogenous Ngb mRNA or protein alterations, effects of selected compounds in Ngb mRNA or protein levels of cultured cell lines and primary neurons need to be examined by quantitative RT-PCR and Western blot for validation; (3) We need to validate the neuroprotective effects of these compounds against neuronal injury induced by OGD or other insults in neuron cultures; (4) Lastly, we need to validate the ability of the selected compounds in up-regulating Ngb in mouse brain and their neuroprotection against cerebral ischemia in mouse models.

We acknowledge that several technological issues require attention in the HTS process. For example, a certain amount of small molecules are interesting during the screening, however, some of them may turn out to be false positives during further validation and optimization, typically after a great deal of time and resources have been devoted, thus it would be critical to minimize false positives during the early screening stages [115]. The HTS process consists of multiple automated steps involving compound handling, liquid transfers, and assay signal capture, all of which unavoidably contribute to systematic variation in the screening data. It will be challenging to distinguish biologically active compounds from assay variability [116]. We may also need to consider building knowledge equity from the integration of multiple parallel screening assays, workstreams and data sources [117]. Lastly, statistical analysis of the screening data may also result in false positives or false negatives, therefore it is essential to apply robust statistical methods effectively and properly for decision-making [115,116,118].

**Figure 2:**
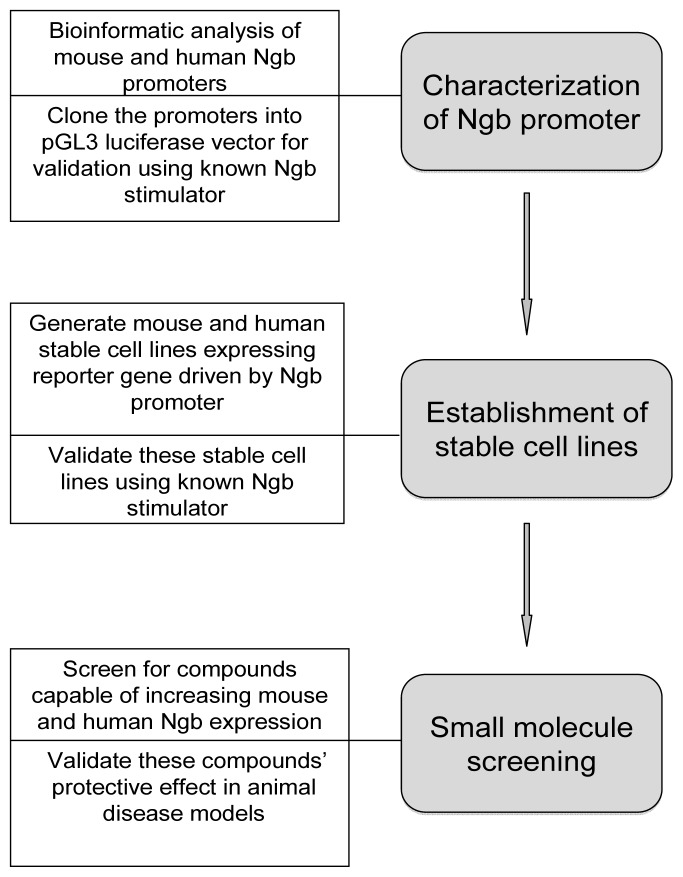
Strategy of small molecule screening for chemical compounds capable of up-regulating Ngb gene expression. The key steps in this strategy include: (**1**) Characterization of Ngb promoter using bioinformatics tools and luciferase reporter construct; (**2**) Establishment of stable cell lines that express reporter genes driven by Ngb promoter; (**3**) Screening and validation of chemical compounds that can up-regulate Ngb expression and be neuroprotective.

In summary, the strategy we described above comprises two translational features. First, we will establish both mouse and human stable cell lines with Ngb promoter reporter to ensure that selected compounds are capable of activating both mouse and human Ngb promoters. Second, validation as the key component will be conducted in every step of small molecule screening and therapeutic development. The general strategy of this small molecule screening system is summarized in Figure 2.
